# A Model for Online Interactive Remote Education for Medical Physics Using the Internet

**DOI:** 10.2196/jmir.5.1.e3

**Published:** 2003-03-31

**Authors:** Milton K Woo, Kwan-Hoong Ng

**Affiliations:** ^1^Toronto-Sunnybrook Regional Cancer CentreDepartment of Medical PhysicsToronto OntarioCanada; ^2^University of TorontoDepartment of Radiation Oncology and Department of Medical BiophysicsToronto OntarioCanada; ^3^University of MalayaDepartment of RadiologyFaculty of MedicineKuala LumpurMalaysia; ^4^University of Malaya Medical CentreKuala LumpurMalaysia

**Keywords:** education, computer uses in education, distance learning

## Abstract

**Background:**

Medical physics is a relatively small community but it spans great geographical distances, usually with a scarcity of experts whose expertise could greatly benefit students entering into the field. In addition there are many software systems for which an interactive education method would be most advantageous.

**Objective:**

To develop a process to optimally use the Internet for real-time interactive remote education of medical physics and to present the experience of the study.

**Methods:**

The project is a collaboration of the Department of Medical Physics at the Toronto-Sunnybrook Regional Cancer Centre in Canada and the Department of Radiology at the University of Malaya in Malaysia. A class of medical-physics graduate students at the University of Malaya attended lectures provided by lecturers in Toronto, using the Internet as the main tool of communication.

**Results:**

The different methods that can be used to provide the real-time interactive remote education were explored, and various topics — including traditional classroom lectures as well as hands-on workshops — were also delivered.

**Conclusions:**

The concept of real-time interactive remote education is viable and holds promise for providing economical and practical tele-education to the medical physics community, but depends heavily on the availability of the Internet in many developing countries.

## Introduction

The Internet has unquestionably become an important aspect in many facets of our daily lives. In the particular area of education, many useful exploitations of the Internet have been in existence for a long time, with courses in many fields being offered online [3].

In the medical physics community, the Internet has already assumed an almost indispensable role, with the dissemination of information and the facilitation of communication [4,5] as well as electronic journals with extensive literature search capabilities, and more recently the cataloging of virtual online seminars from conferences as well as online courses, such as those offered by the American Association of Physicists in Medicine (AAPM) [1].

One aspect that hitherto has not received much attention within the medical physics community is the area of real-time interactive remote education using the Internet, and this is the subject of our present study. This form of real-time education would resemble the common remote classroom-type education, where the video and audio signals of the lecturer are transmitted to a remote classroom and shown on a television set, and the class can pose questions back to the lecturer.

Many factors would favor such a mode of teaching to the medical physics community. Medical physics is a relatively small community, with instructors and students often at great distances apart, and usually with a scarcity of expert teachers. Medical physics services are often lacking but needed in remote areas, including developing countries, and bringing access to expertise to these places would be beneficial. Medical physics also involves many software packages, concepts, and ideas that can only be best taught in a real-time interactive fashion, as well as ideas and information that are time-critical, such as a new protocol or new method of treatment or planning. In addition, a flexible course format allows a particular course to be tailored specifically for a small group of recipients. A simple communication method would also enable experts in the field to provide lectures easily from a convenient location, without the necessity for complicated computer system set-up and support.

The emphasis of the present project then is to investigate the format of real-time remote education that can draw the maximum benefit out of the Internet and benefit the community; in particular aiming towards the medical physics community in developing countries.

## Methods

A collaboration was set up between the Department of Medical Physics at the Toronto-Sunnybrook Regional Cancer Centre in Toronto, Canada and the Department of Radiology at the University of Malaya in Kuala Lumpur, Malaysia, whereby a class of medical physics graduate students at the University of Malaya attended lectures provided by lecturers in Toronto, using the Internet as the main tool of communication. There are two aspects of the project. The first is to experiment with the method with which the real-time interactive communication is provided, as well as the logistics of communication (such as the time-zone difference and availability of the facilities). The second aspect is to determine the optimal contents and formats of such a remote-education project.

A survey of various tools available for tele-education was undertaken. These are summarized in the Appendix and described briefly below. The main "hardware" tools used include a personal computer (PC) at both the instructor's and students' end, with both systems connected to the Internet. In particular, the instructor's computer is connected via a high-speed connection to a local ISP (Internet Service Provider), and the students' computer was connected to the Internet through the university's computer network. Other hardware (such as a regular telephone connection, with a speakerphone) as well as other personal computer accessories (such as a Web camera, microphone and speaker sets, and a drawing tablet) are additional optional hardware that are listed in the Appendix. The software tools include a screen-sharing program by which the class can view the computer screen of the instructor, and hence the lectures could be provided using, for example, PowerPoint slides. The screen-sharing program could also allow the class to interact directly by typing or by drawing on a whiteboard, as well as — with the permission of the instructor — by directly controlling the mouse of the instructor's computer to advance slides or run other programs. Another useful program is one that can provide audio/video communication between the two sides. An alternative to that is to use the regular telephone for audio communication only. As part of the study, many of these tools were tested to determine the usefulness and limitations. The observations are summarized in the Results section.

The curriculum of the course compiles a series of lectures with various topics in radiation therapy. The class consisted of 7 medical physics graduate students who are enrolled in a regular medical physics program at the University of Malaya that covers both imaging and therapy subjects. The topics for the tele-education course were chosen to supplement the regular program, with special attention paid to hands-on demonstration of software packages and to topics requiring more interactive communication between the two sides.

## Results

**Figure 1:**
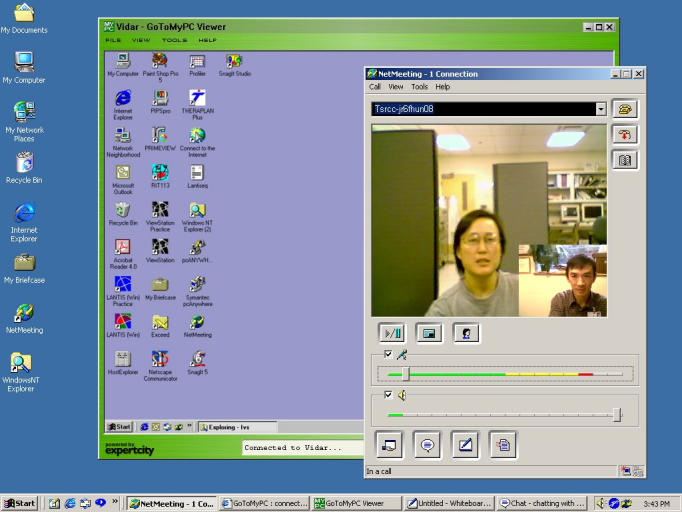
Computer screen at students' PC workstation, showing the shared instructor's PC screen, together with the video screen of both parties

A series of one-hour lectures, including demonstration of software, were given during the course of the project. Figure 1 shows a scenario of the lecture, where the instructor's PC screen appears on the screen of the students' PC, together with the video picture of both parties using the software program NetMeeting [2].

A typical scenario of a lecture proceeded as follows. The details of the lecture, such as the time, the topic, the mode of audio/video communication, would be set up ahead of time via e-mail. At the time of the actual lecture, the instructor would first establish voice communication with the class, via a regular telephone call. The class would then use their personal computer to access the Instructor's computer via the Internet. Once computer communication was established, the class could view the PowerPoint slides that the instructor brought up on his workstation. The lecture would then proceed using the regular telephone network through a speakerphone, as well as the screen-sharing program. The class can interact via the speakerphone as well as the screen-sharing program.

Since this is a pilot project, the students were asked to provide feedback on all aspects of the project. Because of the relatively small number of students involved, the quantitative aspects of the evaluation are not meaningful, but the overall evaluation can be summarized below.

### Time Difference

One of the major difficulties was the 13-hour time difference between the two locations. This greatly affected the testing of the hardware and software tools, as well as the organizational aspect of the actual lectures. It was most practical for the instructor to deliver the lecture from home in the evening, using a laptop computer connected to a high-speed Internet service provider. The students would then attend the class at the university during the morning. The times had to be reversed for some of the workshops where the software was not available on the laptop computer, and so the instructor demonstrated the software at the office in the morning, while the students held the class at the University in the evening. Once the students were familiar with the software, they were allowed to log into the instructor's office computer using a guest account and run the software program independently. The students also managed to download the PowerPoint slides for offline viewing.

### Communication Method

The preparation for the actual lectures involved experimentation with and the analysis of the different communication facilities and methods. These are summarized in the Appendix. The configuration we found most reliable in carrying out the series of lectures is:

#### Availability of the Internet

The speed of the response depended critically on the traffic on the Internet network, which could vary significantly with the time of day. When the lectures were given in the evening in Malaysia the response was acceptable, with a screen refresh usually transmitted to the students' computer within 10 seconds.

#### Screen-sharing Software

The screen-sharing system GoToMyPC [6] was used as the tool for the lecture presentation. This system requires a subscription to the service provider, after which a series of instructions on the Web site allows the easy installation of a program on the host computer. The students then go to the same Web site and follow some simple instructions to access the instructor's computer. This software was the most suitable one for our requirements because of its ease of use, its ability to work properly in the presence of firewalls, and its relatively low cost.

#### Audio Communication

Effective audio communication was critical to the success of the lectures. Using the regular telephone network was most reliable, and the cost was relatively low.

#### Video Communication

The video communication using the computer software NetMeeting as depicted in Figure 1 was also attempted but because of the firewall problem was not successful; but is still being pursued. The lectures managed quite well without video communication.

#### Firewall

The presence of the firewall proved to be a major hurdle that prevented the use of many software tools. The simple combination of GoToMyPC and the telephone proved to be sufficient to allow us to complete a series of one-hour lectures.

### Course Contents

A standard medical physics curriculum already exists at the medical physics department at the University of Malaya, so a series of special topics, such as IMRT (Intensity Modulated Radiation Therapy), was selected for the lectures. A demonstration of a treatment-planning system was also carried out. It was found that lectures that can be given using PowerPoint slides were most suitable for this tele-education project.

### Class and Instructor Interest

The class welcomed the opportunity to interact directly with experts in the field. The relatively-small community of medical physics would favor small class sizes and interactions, as well as the involvement of experts at distant locations.

### Cost

It is encouraging to report that the cost of the remote-education program could be kept to quite a bargain-level. This, of course, depends on the availability of fast Internet access. The software program we used, for example, costs about US $100 per year to install on the instructor's computer. The other costs such as telephone costs and minor hardware items, are insignificant compared to, for example, travel costs to workshops. The major cost that remains would be the lecturer's fees. Since the process offers the lecturer a great deal of flexibility in terms of time and place for the lectures, the "cost" to the lecturer is kept to a minimum. In addition, this is a new and exciting process that many will find interesting to participate in. Hopefully this will encourage lecturers to donate their time for an affordable and worthwhile project.

To start a real-time interactive remote education program, however, careful planning must be undertaken. In a recent case study on using the Internet to teach health informatics [7], various technical difficulties, in particular for synchronous communication, were discussed. Computer and network problems could be very frustrating and minor glitches could discourage both the instructors and students. It is highly recommended that a new program be carried out with a minimal but reliable set of tools, such as the standard telephone used in our project, and preferably with the support of knowledgeable computer consultants.

The issue of whether the Internet is a net disservice to the medical physics community in developing countries is a recent debate topic [8]. Hopefully with the increased availability of high-speed Internet service, a remote-education program like the one reported here can bring practical benefits to the developing countries.

## Discussion

### Video Communication

Although the lectures managed quite well without video communication, it is felt that its addition would have provided a valuable feedback mechanism.

### Firewall

Although the simple combination of GoToMyPC and the telephone proved to be sufficient, it is felt that if a connection could be established without the blockage of the firewall then the method of communication could be more flexible and powerful. Because of this, it is recommended that the computer department in the organization or the university be involved if a firewall exists.

### Instructors

Instructors could give lectures at any convenient location where there is access to the Internet, and the screen-sharing software used for the project could be uninstalled from one PC and reinstalled on the guest-lecturer's PC easily and legally.

### Course Contents

Topics such as dose calculation would require more blackboard-type illustrations and are more difficult to carry out, although if a drawing tablet were available the process would have been easier. A particular advantage of this mode of tele-education is that very-specialized expertise could easily become accessible in otherwise impractical situations.

### Conclusions

In conclusion we feel that the project proves that real-time interactive remote education of medical physics is a viable concept that is ready to be carried out with selected places and groups. It serves a useful purpose and is a very cost-effective way to promote closer communication and to spread knowledge and information within the medical physics community. There are certainly limitations and any new programs must be carefully planned. The increased usage of the method will speedily eliminate some of the existing limitations.

## Abbreviations

ISP: Internet Service Provider
PC: Personal Computer

## Appendix 1

Screen-sharing Software
                        **Operational Characteristics:**
                    A screen-sharing program allows the students to "share" the instructor's PC screen so they can view what the instructor brings up on the screen (such as PowerPoint slides or a treatment-planning system), and could also be given authorization to execute commands on the instructor's PC (such as to run application programs or pose questions to the instructor).
                        **Examples:**
                    GoToMyPC https://www.gotomypc.com/
                    Windows XP http://www.microsoft.com/windowsxp/default.asp
                    PCAnywhere http://www.symantec.com/pcanywhere/index.html
                    Remote Administrator http://www.radmin.com/default.html
                    VNC http://www.uk.research.att.com/vnc/index.html
                    WEBEX http://www.webex.com/

                        **Factors to Consider:**

                        ***Speed***
                    The software module works by sending an image of the instructor's PC screen over the Internet. Some compression is used and often only an image of the part of the screen that is updated is sent. The main determining factor is the speed of the Internet traffic, which could be quite variable. For a simple PowerPoint lecture with no images to transfer, a dial-up line at 56K isadequate. The demand is much more severe for interactive screen sharing, where the student controls the instructor's PC. In this case the keystroke has to be transmitted, and the delay of the typed character is noticeable even with the fastest Internet connection,and both sides have to adjust to this delay. The response speed could also depend significantly on theparticular software.
                        ***Cost***
                    This could vary significantly with different pieces of software,with some charging a one-time cost and others by usage. Some require a license on each PC, while others require an annual feefor each host (ie, instructor's PC), etc.
                        ***Firewall Compatibility***
                    The firewall in the computer network of most institutions pose a problem for most screen-sharing software, and the Information Systems department has to be involved to allow these software programs to work.
                        ***Features***
                    Some of the Web-conferencing packages include many tools other than screen sharing, such as audio/video, whiteboard. A particularly useful feature is to allow multiple sites for both the students and lecturers to be online concurrently.
                        ***Ease of Installation and Use***
                    Some software programs have to be installed on both sides, while others can be accessed directly through the Internet browser. Many of the Web-conferencing packages require fairly complicated setups, especially on the instructor's end.

Audio
                        **Operational Characteristics:**
                    Audio communication is essential in delivering the lectures remotely. The regular telephone is probably the most straightforward means, but with the increasing availability of voice communication over the Internet, using the PC for audio communication offers certain advantages.
                        **Examples of Audio Communication Alternatives:**
                    Software is available to allow voice communication using PCs. Both the Instructor and the students could have software to enable voice communication. Alternatively, either side could use a regular telephone while the other party uses the PC. Speakers and microphone attachments are readily available on PCs.
                        **Factors to Consider:**

                        ***Cost***
                    The cost of long distance telephone calls could vary significantly depending on the carrier used but could remain quite reasonable for providing this type of remote education. For PC-to-PC communication there is often no extra charge other than the Internet charges. Not having to deal with telephone charges, however small, is a convenience especially when guest lecturers are invited to participate.
                        ***Availability***
                    A regular telephone line may not be always available especially when it needs to be dedicated for the duration of the lecture. A speakerphone is sometimes more difficult to set up than the soundsystem for the PCs. Being able to integrate all the functions into the PCs is a nice feature. However, the firewall again poses a major problem for audio and video communications.
                        ***Quality of Audio Communication***
                    The audio quality on the regular telephone network is still superb compared to that over the Internet, although the delay and echo on international telephone calls is still noticeable at times.This delay is inevitably noticeable when using audio communication over the Internet, although it is improving and with practice the quality is quite acceptable.
                        ***Ease of use and installation***
                    The regular telephone is probably the most straightforward to use although as mentioned a speakerphone may not be readily available. PC-based audio communication requires setup but is usually not too complicated.

Video
                        **Operational Characteristics:**
                    Relatively inexpensive cameras (Web cams) are often available to enable real-time video images to be sent and received over the Internet. The video display can be displayed simultaneously with the Instructor's screen (Figure 1) or could be minimized.
                        **Examples of Video Communication Software:**
                    Many Web-conference software programs mentioned in Screen-sharing Software (above) incorporate both audio and video features. Alternatively, there are independent software packages that could be run in parallel with the screen-sharing software.
                        **Factors to consider:**

                        ***Quality of picture***
                    Because of the large amount of data required for video communication, the quality is quite poor (eg, NetMeeting has a resolution of only 176 pixels x 144 pixels). The delay and discontinuity of the image transmission is quite obvious, although with high speed Internet the quality is better. Despite the relatively low quality of the video transmission, the images are still quite acceptable and serve as a valuable means of feedback.
                        ***Cost***
                    This is relatively low, with the software usually included in the Windows operating system, and the Web cam is the only cost.
                        ***Other factors***
                    Video is also useful for quickly showing images not available through the screen-sharing program, eg, a piece of equipment. Video communication is not essential, and a remote lecture could indeed be carried out without it. This could definitely be an issue if the student site does not have the funding or expertise for a complicated computer set-up.

Other Options Other options such as whiteboard, overhead transparencies, and movie capabilities, may help to enhance the quality of thelecture. The Web cam mentioned above could be used to show hand-drawn diagrams, but for extensive blackboard-type lectures a drawing tablet may be useful in conjunction with the screen-sharing program. Similarly, the Web cam can be used to show overhead pages, or actual procedures or equipment.

Networks These could include dedicated networks such as ISDN (Integrated Services Digital Network), which allows direct connection of the lecturer and students. The advantages are more-reliable connection and fast response. The other alternative is to use the Internet in which a connection may be switched over many routes. The disadvantages are the dependability of communication speed and reliability on the network traffic. The big advantage however is the wide and ever-increasing availability of the Internet and with that, increased reliability.
